# Social Network Size in the Years Before Death: An Examination by Dementia Status

**DOI:** 10.1093/geroni/igaf122.1165

**Published:** 2025-12-31

**Authors:** Sara Moorman, Alyssa Goldman

**Affiliations:** Boston College, Chestnut Hill, Massachusetts, United States; Boston College, Chestnut Hill, Massachusetts, United States

## Abstract

There is growing recognition that in addition to between-person differences in the size of older adults’ social networks, within-person network growth or shrinkage also influence well-being. Relatively little attention has been paid to patterns of social network change at the end of life, when health declines can render social networks especially important for quality of life, while also potentially compromising individuals’ ability to maintain social ties. Using longitudinal data from respondents of the National Health and Aging Trends Study who have died, we examine how core discussion network size changes in the years before death, with particular attention to differences in cognitive status at the end of life. Tests of mean differences indicated that respondents with normal cognition in the last interview before death had significantly larger core discussion networks (M = 2.10) compared to those with probable dementia (M = 1.79; p< .001). Among respondents with normal cognition in the last interview (N = 1,642), growth curve models revealed a linear decline of 0.03 social network members per year in the years before death (p<.001). Among respondents with probable dementia in their last interview (N = 1,002), network size did not change significantly in the years before death. Our results suggest that effective efforts to support older adults’ social engagement may depend on both timing and cognitive health status. Social interventions may provide more benefits for those with dementia if implemented before the end-of-life period, while efforts implemented at the end of life may still benefit older adults with normal cognition.

